# Power Up: Combining Behavior Monitoring Software with Business Intelligence Tools to Enhance Proactive Animal Welfare Reporting

**DOI:** 10.3390/ani12131606

**Published:** 2022-06-22

**Authors:** Jason David Wark

**Affiliations:** Animal Welfare Science Program, Lincoln Park Zoo, Chicago, IL 60614, USA; jwark@lpzoo.org

**Keywords:** animal welfare, behavior monitoring, business intelligence, evidence-based decision making, data visualization

## Abstract

**Simple Summary:**

Monitoring animal behavior over time is important for zoos and aquariums seeking to continually evaluate animal welfare. Although new digital tools are making behavior monitoring more accessible, analyzing behavior data in a timely manner to draw meaningful insights can be challenging. Business intelligence software has the potential to help address these challenges. Business intelligence software is a class of tools that combines the ability to integrate multiple data streams with advanced analytics and robust data visualizations. Here, I highlight features of the Microsoft Power BI platform as an example. Power BI is a leading option in business intelligence software and is freely available. To demonstrate the potential of business intelligence tools for behavior monitoring, I provide two example data dashboards of data recorded using the ZooMonitor behavior recording software. The first dashboard illustrates a simple quarterly behavior summary to track behavior changes in an ongoing manner. The second dashboard visualizes data relating to enrichment evaluation. I hope this introduction to business intelligence software and the Microsoft Power BI platform can provide researchers and managers in zoos and aquariums with new tools to support their evidence-based decision-making processes.

**Abstract:**

Animal welfare is a dynamic process, and its evaluation must be similarly dynamic. The development of ongoing behavior monitoring programs in zoos and aquariums is a valuable tool for identifying meaningful changes in behavior and allows proactive animal management. However, analyzing observational behavior data in an ongoing manner introduces unique challenges compared with traditional hypothesis-driven studies of behavior over fixed time periods. Here, I introduce business intelligence software as a potential solution. Business intelligence software combines the ability to integrate multiple data streams with advanced analytics and robust data visualizations. As an example, I provide an overview of the Microsoft Power BI platform, a leading option in business intelligence software that is freely available. With Power BI, users can apply data cleaning and shaping in a stepwise fashion, then build dashboards using a library of visualizations through a drag-and-drop interface. I share two examples of data dashboards built with Power BI using data from the ZooMonitor behavior recording app: a quarterly behavior summary and an enrichment evaluation summary. I hope this introduction to business intelligence software and Microsoft Power BI empowers researchers and managers working in zoos and aquariums with new tools to enhance their evidence-based decision-making processes.

## 1. Introduction

Increased attention toward animal welfare, likely driven by both ethical and regulatory reasons, has made monitoring animal welfare a fundamental task for many animal-related organizations [1]. Although many definitions of animal welfare exist [2,3,4,5], there is general agreement that welfare can vary along a continuum and can change over time. Thus, welfare can be viewed as a dynamic process [6]. For applied welfare practitioners in zoos, aquariums, and laboratories, evaluating welfare is often done in an effort to drive husbandry changes that can enhance welfare. These actions then lead to a need for additional welfare evaluations. As welfare is a dynamic process, our methods to assess welfare must be equally dynamic. Ultimately, zoos and aquariums require systems for monitoring welfare to be dynamic and support evidence-based decision making.

Behavioral monitoring programs, whereby animal behavior is systematically documented in an ongoing manner, are an attractive approach to evaluating animal welfare [7]. The importance of animal behavior as an indicator of welfare is well-recognized [3], and behavior has been shown to be the most commonly assessed measure of welfare [8]. Unlike traditional hypothesis-driven animal behavior research, where behavior is typically recorded during a fixed study period, often with experimental treatments, behavioral monitoring programs seek to continually monitor behavior over time to establish individual baseline behavioral patterns. Trends and deviations in these patterns can then be monitored to proactively identify potentially meaningful changes to welfare. Recently, new digital tools have simplified behavior data collection, allowing users to quickly record the behavior of animals using mobile devices [9]. Although these new tools have streamlined data collection, they have not addressed many of the inherent challenges of quickly analyzing large amounts of data and reporting findings as necessitated by a dynamic welfare monitoring process.

Analysis and reporting of data from behavioral monitoring programs differs from traditional hypothesis-driven research in several important ways. The most fundamental difference is that the data from behavioral monitoring programs are ongoing without a defined study period, after which time data would be analyzed. Furthermore, behavioral monitoring programs may start without a clear question to guide analyses. In addition, behavioral monitoring programs often focus on applied decision making, with a need for quick insights that can be put into action. Because of the lack of guiding questions and the need for timely data analysis, the statistical approach can differ between both types of data collection, with behavioral monitoring programs often relying more heavily on exploratory data analysis to identify meaningful patterns, in contrast with the inferential analyses that are common to traditional animal behavior research. Lastly, there is a key difference in how the results are shared, with behavioral monitoring programs typically generating reports that are shared internally at the organization with managers, whereas traditional research is typically shared through peer-reviewed publications and presentations at scientific conferences. Therefore, applied animal welfare monitoring often requires different analysis tools than are typically used in traditional research to address these unique analytics needs.

In this paper, I introduce potential solutions in the field of business intelligence (BI) software. I first provide a brief overview of the history of BI and its modern usage. Next, I highlight an accessible BI option as an example, Microsoft Power BI. Lastly, I illustrate how behavioral data from the ZooMonitor behavioral monitoring software can integrate with Microsoft Power BI to provide robust analytics for applied welfare monitoring. The goal of this paper is to familiarize managers and scientists looking to closely track animal welfare, who may currently rely on spreadsheet applications like Microsoft Excel for their BI needs, with available BI solutions they can implement to inform their decision-making processes. Although a step-by-step tutorial is beyond the scope of this paper, I aim to provide the reader with a sufficient background on the key concepts that are necessary for getting started with BI software. The adoption of new tools can require a large investment in time and energy and may be daunting. I hope to convince the reader of the transformational potential that these accessible BI tools can have in maximizing the impact of our monitoring efforts on the welfare of animals.

## 2. Business Intelligence

### 2.1. Defining “Business Intelligence”

The term “business intelligence” is believed to have been first recorded by Richard Miller Devens in 1865, used to describe how a banker was adept at tracking news to exploit current events for personal and financial gain [10]. Nearly 100 years later, in 1958, Hans-Peter Luhn subsequently used the term when proposing a system that could scan documents to extract and analyze text to gain “business intelligence” [11]. Our modern view of BI is typically credited to Howard Dressner, who, in 1989, coined BI as an umbrella term to describe the growing assemblage of computerized technologies that helped to make “decisions based on fact” [12]. A popular modern definition of BI is that of Wixom and Watson [13]: “Business intelligence is a broad category of technologies, applications, and processes for gathering, storing, accessing, and analyzing data to help its users make better decisions.” Although our modern views of BI have evolved greatly and extended beyond traditional business contexts (e.g., health care [14,15,16], and education [17,18]), the fundamental concept of analyzing information, often from various sources, to make an informed decision, has remained.

**Figure 1:**
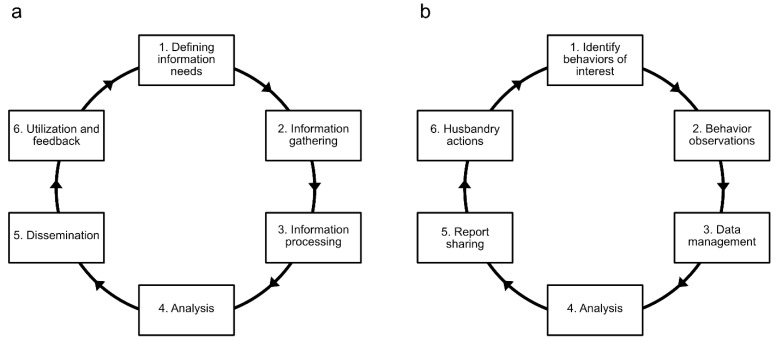
A diagram of the steps involved in (**a**) the business intelligence process as described by Vuori [21], and (**b**) a behavior monitoring program.

Given the interdisciplinary nature of BI, it is therefore not surprising that it can be viewed from many perspectives [19]. At a broad level, BI can be viewed as a type of information system, a term used to describe the integrated organization and actions surrounding the collecting, storing, and processing of data, and the distribution of resulting information [19]. Similarly, it has also been described as a type of data-driven decision support system [20]. In addition to these organizational perspectives of BI, it has also been described in more data-centric terms. For instance, Vuori [21] presented BI as a cyclical process, with questions leading to data and insights that then create more questions (Figure 1a). This view of BI as a cyclical process draws an immediate comparison with the steps of behavior monitoring programs, whereby initial behaviors of interest for observation are identified that can then be subsequently impacted by husbandry actions and necessitate continued monitoring (Figure 1b). 

### 2.2. Modern BI Tools

Commonly, the term BI is used to refer to a special class of advanced analytics software. Chaudhuri et al. [22] defined BI software as the “collection of decision support technologies for the enterprise aimed at enabling knowledge workers such as executives, managers, and analysts to make better and faster decisions”. Many in applied welfare roles are likely to rely on spreadsheet programs like Microsoft Excel for their BI needs. Although Microsoft Excel may qualify as a traditional BI solution, many modern BI tools have emerged in recent years, designed to provide robust reporting and analytics options. These new tools may better support applied welfare practitioners seeking to gain quick insights from their data that can be used to guide husbandry actions.

Each year, Gartner Research evaluates the available analytics and BI platforms on the completeness of their offerings. In their 2022 report, Gartner Research defined 12 critical capabilities for a modern BI platform, including security, governance, cloud-enabled analytics, data-source connectivity, data preparation, catalogs, automated insights, data visualization, natural language query, data storytelling, natural language generation, and reporting [23]. Based on these criteria, Gartner Research highlighted three companies as leaders in their Magic Quadrant report: Microsoft for their Power BI platform, Salesforce for their Tableau software, and Qlik for their analytics platform. Microsoft’s Power BI platform has been selected as a leader in analytics and business intelligence for 15 consecutive years [24]. Given this strong dominance in the field of BI software and the ubiquity and accessibility of Microsoft’s platform, I highlight key features of the Microsoft Power BI software for readers who are unfamiliar with this BI tool.

## 3. Overview of the Microsoft Power BI Platform

The Microsoft Power BI software originated out of several advanced add-in features of Microsoft Excel, namely the Power Query tools for importing and shaping data, the Power Pivot engine for modeling data, and the Power View feature for creating interactive visualizations. These tools were organized into the Power BI platform and made publicly available in 2015, although Power Query and Power Pivot are still available in recent versions of Microsoft Excel (development for Power View ended in 2016). Microsoft Power BI is available in three different versions: (1) Power BI Desktop—a desktop app designed primarily for creating reports; (2) Power BI service—a cloud-based SaaS (Software as a Service) component intended for collaboration and alerting; and (3) Power BI mobile apps—iOS and Android apps for viewing reports and dashboards on-the-go. Reports created in Power BI Desktop, a free application for download, would typically be published to the Power BI service, where they can then be shared with other users with Pro or Premium licenses, and made available in the Power BI mobile apps. I provide a brief overview of the key features below and how they relate to behavioral monitoring programs. For a more in-depth guide to the Power BI platform, I recommend the reader to view Microsoft’s online training tools [25] and Ferrari and Russo [26].

### 3.1. Reports and Dashboards

**Figure 2:**
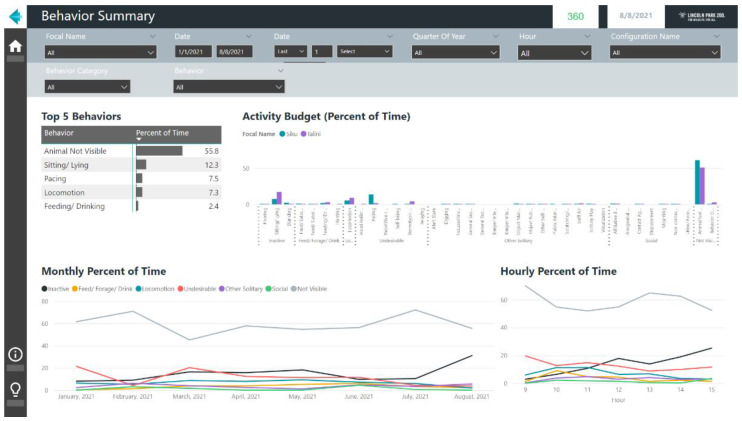
An example screenshot of a report in the Microsoft Power BI software.

As the terms *report* and *dashboard* in Power BI may be confusing to new users, I provide a brief description. In Power BI, the term report is used to refer to interactive dashboards that users can create by dragging and dropping different data visuals (e.g., column charts, line charts, etc.) onto a blank canvas. These reports are typically created in the Power BI desktop application but may also be created through the Power BI service. Reports can feature multiple pages, shown as tabs in the Power BI interface. Navigational controls, such as back buttons and links to pages, can be added to create a fully interactive experience. An example page of a Power BI report highlighting the general behavior patterns in polar bears housed at Lincoln Park Zoo is shown in Figure 2.

**Figure 3:**
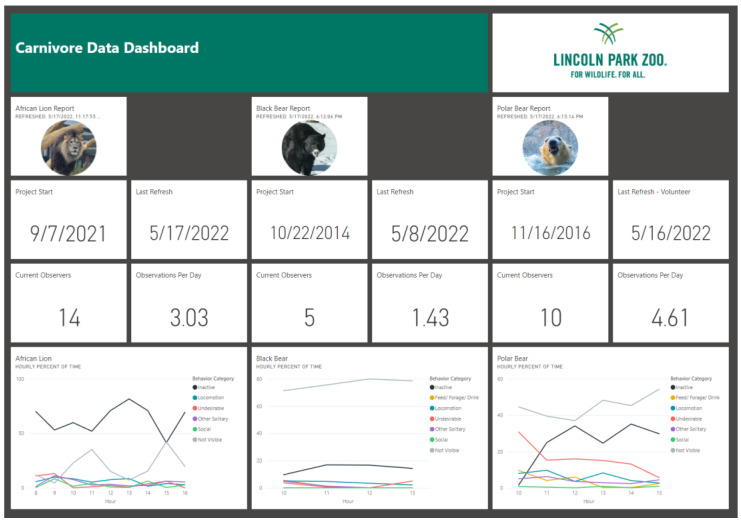
An example screenshot of a dashboard in the Microsoft Power BI software.

In contrast, dashboards in Power BI refer to a page that the user can create in the Power BI service, where they can pin items from different reports that have been published to the Power BI service, including individual data visuals, links to report pages, or the entire report pages. These items appear as tiles on the dashboard that can be resized and rearranged. Dashboards are useful for quickly monitoring important metrics across multiple reports in a single area and easily navigating between the underlying reports. An example of a Power BI dashboard highlighting data from several carnivore species is shown in Figure 3.

### 3.2. Data Sources

Power BI supports a wide variety of data sources, including text and CSV files, Excel workbooks, HTML web tables, and many different database types [27]. In addition, Power BI reports can also specify a folder as a data source to import multiple files of the same type simultaneously. This can be particularly helpful when working with data from sensors or camera systems that may generate a large number of files. For users of Microsoft’s OneDrive cloud storage, the files can also be referenced using web links to access the data stored in the cloud.

### 3.3. Data Modeling

Data in Power BI, like many other modern BI tools, are stored in relational databases. Although these types of databases are not unique to Power BI, a basic understanding of their structure and function is important for getting started with Power BI. In contrast to spreadsheet applications, where data are stored in a single table with individual cells that can be manipulated, data in a relational database can be stored across multiple tables that are referenced by columns (fields) and rows. The fields in these tables are then related to one another through defined relationships, such as a one-to-many relationship where a field in one table with unique values is related to a field in another table with duplicate values. A simple example of this could be between the behaviors in an ethogram, the list of defined behaviors for a study, and the recorded behaviors stored in a raw data table. Each behavior in the ethogram is unique but may be recorded multiple times during behavior observations and thus may occur multiple times in the data table. These unique fields that are used to relate multiple tables are typically referred to as keys [26]. In addition to the one-to-many relationship, Power BI also supports many-to-one, one-to-one, and many-to-many. Each of these relationships can then be filtered in a single direction, whereby a key in one table is used to filter another table, or in both directions, where the field in either table can be used to filter the other table.

When a relational database contains more than two tables, it is important to consider how all of the tables relate to each other. The collection of tables with their relationships is described as a “data model” and can be viewed in Power BI through the Model view [26]. The Model view displays a diagram of all tables and their included fields, with lines between fields in different tables indicating their defined relationships. Each relationship line features endpoints with the number one or an asterisk to indicate the one or many side of the table relationship. The relationship line also has a single or bi-directional arrow to represent the directionality of filtering between the two linked tables. By default, Power BI will automatically search for relationships when data are loaded by identifying shared fields across tables.

Although Power BI will automatically define relationships, understanding the data model and reviewing it as needed is important as logic conflicts may occur with improperly specified relationships. For instance, using the previous ethogram example, an additional table for the behavior category (e.g., inactive behaviors, locomotor behaviors, social behaviors, etc.) could be included. The behavior category table and ethogram table would likely have a one-to-many relationship, as there would be multiple behaviors within each category. However, if a relationship between the behavior category table and the raw data table were defined, this could create ambiguity, as the behavior category table could then filter the raw data table from multiple directions (e.g., directly from the behavior category table, or through the ethogram table). Although there are many different ways that you can define your model, one common and recommended approach is a “star” schema that features relationships radiating out (like a star) from a central table that is typically your raw data table [26]. For a more detailed discussion on data modeling in Power BI, I recommend Ferrari and Russo’s accessible introduction to the topic [26].

### 3.4. Data Shaping

In Power BI, data can be arranged and shaped through the Power Query editor that is accessed through the Transform Data button. This Power Query editor gives users a front-end interface for the “M” scripting language. Using this interface or through a text file with “M” code, data can be manipulated through individual steps. This series of transformation steps for a given data source is referred to as a *query*. The stepwise process for query building can make it easy to troubleshoot errors, as the user can check the results of each step separately.

Power BI offers many options to manipulate data, including common functions to pivot or unpivot data, merge or split columns, or group rows on a given value, to name a few. Additional columns can also be added in multiple ways, including using custom formulas to manipulate the data, or based on conditional formula steps. For example, users could easily bin temperature readings into ranges for analysis, or group individuals by age classes. The ease of specifying conditional logic in a point-and-click interface may be particularly valuable for some as a user-friendly alternative to the formula logic that is necessary in other tools like Microsoft Excel. For users that are familiar with the R or Python scripting languages, text files with data transformation steps can also be referenced and applied through the Power Query editor.

### 3.5. Visualizations and Advanced Analytics

Data visualizations are the foundation of BI tools as they can provide decision-makers with an at-a-glance overview of key patterns. In zoos and aquariums, clear and timely visualizations are crucial for animal managers seeking to use data to help identify and respond to any behavior changes that may reflect a change in welfare. Building a report in Power BI is accomplished through a drag-and-drop interface by selecting a data visual from the library and placing it on the report canvas. Power BI’s chart library has similar basic built-in data visualizations to Excel, including common charts like column charts, line charts, and pie charts. In addition to these basic charts, Power BI also has data visuals designed to show a single metric, such as cards where you can display a value and can conditionally format the card to highlight when values are above or below a set value.

**Figure 4:**
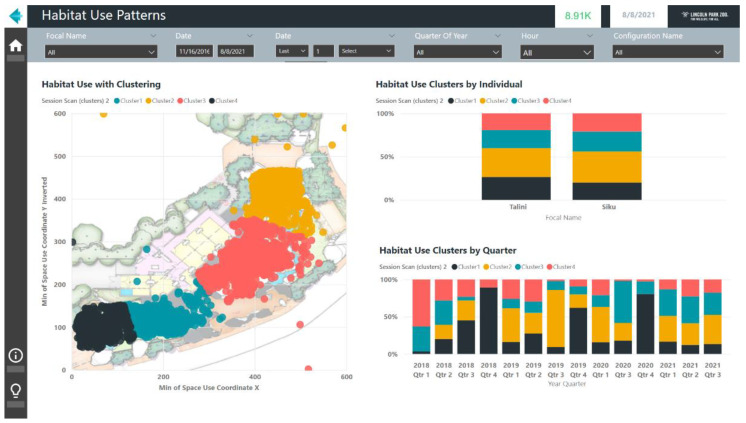
An example screenshot of a report showing habitat use data for two polar bears clustered using built-in algorithms in the Power BI software.

For numerical data, the scatter chart includes built-in tools to help analyze the data for patterns. Scatter chart data can be automatically clustered using Power BI’s built-in algorithms, which will automatically identify groupings in the X and Y coordinate values of the scatterplot based on their numerical values and create new variables for the resulting clusters. These clusters can then be used when creating other charts. For example, in Figure 4, the XY coordinate data reflecting the habitat use of two polar bears at Lincoln Park Zoo were plotted and four clusters in their habitat use were identified. These clusters can then be compared between the two polar bears to view their individual variation and can be compared across calendar quarters to view the changes over time. Binning spatial data in this manner through clustering may provide a less biased view of space use than alternative strategies that require defining zones a priori and, therefore, may more closely reflect the animals’ perceptions of their space. For more advanced visuals, users that are familiar with the R or Python scripting languages can also add charts built from those graphing libraries. In addition to these built-in options for data visuals, Microsoft has also made open-source visuals possible in the Power BI platform. There is a marketplace with both free and paid third party visuals available to download and use.

### 3.6. Sharing and Alerts

Power BI reports and dashboards can be shared in several ways. Reports can be saved as PDF files from the desktop application and shared directly. In addition, users can publish reports to the Power BI service, where they can also export reports as PDF files or embed them as interactive slides in PowerPoint slideshows. In the Power BI service, reports can be shared directly with other Power BI users within an organization or added to shared dashboards to provide key stakeholders, such as animal managers in zoos and aquariums, with an up-to-date view of the data. Sharing reports and dashboards in the Power BI service requires a paid Power BI Pro or Premium license. In the Power BI service, alerts can also be configured to trigger an email to be sent when a specified value exceeds a set range. To create alerts, a card tile displaying the metric of interest must first be pinned to a dashboard and an alert can then be added by setting a threshold limit. In addition to alerts, subscriptions to dashboards and reports are also possible, allowing an email to be sent at a frequency that is customized by the user.

## 4. Integration of Behavior Monitoring Software and BI Tools

Given the functionality of BI tools, this makes them an attractive option for leveraging data from behavioral monitoring software to build at-a-glance insights and inform decisions. Since its release in 2016, the ZooMonitor app has become a leading behavioral monitoring tool in zoos and aquariums, with over 350 active accounts across 40 countries. Developed by Lincoln Park Zoo and Zier Niemann Consulting, ZooMonitor enables the recording of behavior and space use data during live observations using common recording methods. ZooMonitor is freely accessible for accredited zoos, aquariums, sanctuaries, and museums, and is available at a low cost for others. With ZooMonitor, data can be recorded offline on tablet devices, then uploaded to a secure cloud database. ZooMonitor offers built-in reports to allow users to visualize their data, including a behavior budget report to identify broad patterns in the average time spent in different behaviors, a trend graph to see changes over time, and a heatmap to visualize space use patterns. Although these built-in reports can be valuable for answering basic behavior questions (e.g., how much time animals are engaged in a given behavior, where they spend their time, etc.), they may be limited in their ability to address more complex questions that naturally arise during ongoing behavioral monitoring (e.g., is a behavior occurring more frequently after a husbandry change, do animals use their space differently across seasons?). To address these scenarios and others common to applied welfare practitioners, data can be exported from ZooMonitor as a comma-separated-values (CSV) file and imported to Power BI for creating custom behavior reports. To help illustrate the potential of integrating ZooMonitor data with Microsoft Power BI, I share two examples below: a quarterly behavior summary report and an enrichment evaluation report.

### 4.1. Example Microsoft Power BI Report–Quarterly Behavior Summary

**Figure 5:**
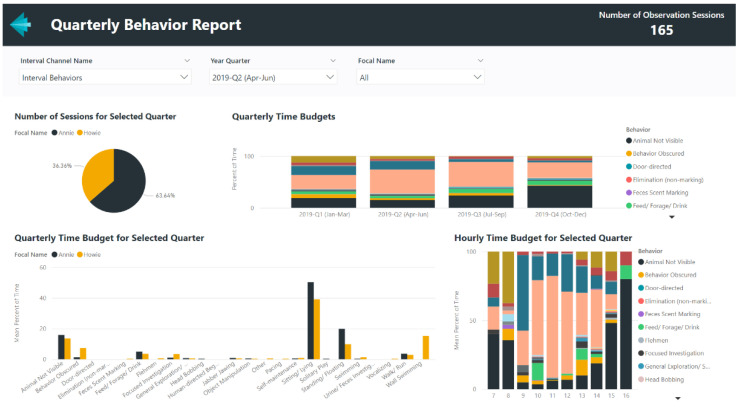
An example screenshot of a report showing quarterly behavior patterns created in Power BI using data from the ZooMonitor behavior recording app (the report template is available for download, see Supplementary Materials File S1).

For behavior monitoring programs, routinely summarizing ongoing data can help to proactively identify meaningful behavior changes. With traditional spreadsheet programs like Microsoft Excel, these recurring reports can be time-consuming and, depending on the analyst’s workflow, may require refreshing the data for each visual and transferring graphs to a text document. In Power BI, a single refresh of a report will update all visuals and their underlying datasets, potentially saving time and allowing the analyst to focus on interpreting behavioral changes instead of data management. Data from ZooMonitor will first need to be exported and stored in a centralized location (e.g., a master Excel file), as direct integrations between ZooMonitor and Power BI are currently not available. An example Power BI report displaying a quarterly summary of behavior patterns is shown in Figure 5. This report was created using data from the ZooMonitor app and the template that is available to download and use with individual animal data from any ZooMonitor project (see Supplementary Materials Files S1 and S2). This report uses behavior data exported from the ZooMonitor app and features a separate page for each recording method in ZooMonitor: interval, continuous, and all occurrences [28]. As the ZooMonitor app makes it possible to create custom projects tailored to specific species or questions, the user must first select in the report the recording channel to visualize.

### 4.2. Example Microsoft Power BI Report–Enrichment Evaluation

**Figure 6:**
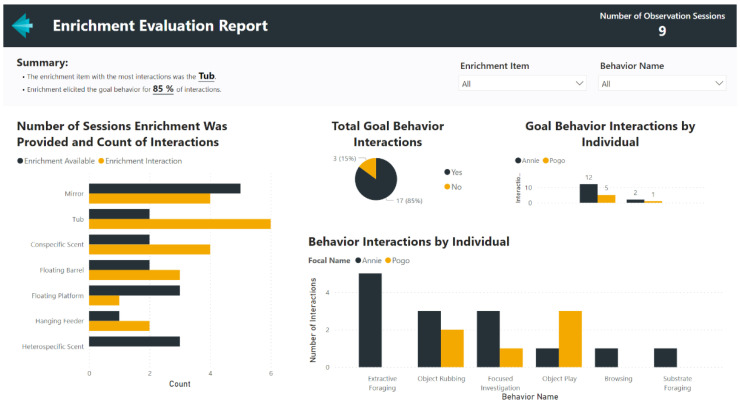
An example screenshot of an enrichment evaluation report created in Power BI using data from the ZooMonitor behavior recording app (the report template is available for download, see Supplementary Materials File S2).

In modern accredited animal facilities, providing environmental enrichment to animals is an essential daily activity for animal care staff [29]. Often intended to promote natural behaviors, the ultimate success of enrichment towards this goal can be difficult to evaluate from daily animal keeper records [30]. Frequent, brief behavior observations have been shown to be an effective method for evaluating enrichment [31,32], suggesting that these challenges in evaluation could be addressed through behavior monitoring programs. An example Power BI report to evaluate enrichment using the ZooMonitor app is shown in Figure 6. This report is available as a template to download, along with example data and instructions for use (Supplementary Materials Files S3–S5). This Power BI report was built using data from a ZooMonitor project that included two behaviors that could be easily added to any ongoing monitoring project: *Enrichment Available* and *Enrichment Interaction*. When starting an observation session, the observer can select the Enrichment Available behavior to record the enrichment items present during the observation. When the animal interacts with an enrichment item, the observer can select the Enrichment Interaction behavior and record the enrichment item that elicited the behavior, the specific behavior, and whether the elicited behavior was a goal for the enrichment item.

### 4.3. Incorporating Additional Data Sources to Enhance Reporting

In these previous examples, I highlighted ways you could enhance reporting with Microsoft Power BI using the ZooMonitor exported data as a single data source. However, some behavior questions may be complex and require data across multiple platforms. With Power BI, data from ZooMonitor could be combined with other sources in a single report. Additional data sources that may be particularly valuable include: dataloggers, animal records management systems, and customer relations management (CRM) platforms. These sources typically offer non-proprietary data formats for exporting and, in some cases, it may be possible to import data directly from the software database (e.g., Salesforce data connector in Power BI). Connecting these sources could allow for robust behavior insights. For example, seasonal changes in behavior could be evaluated by combining behavior data (ZooMonitor) with data on the temperature (dataloggers), animal weights (records management system), and daily guest attendance numbers (CRM). Although these sources could be combined in familiar tools like Microsoft Excel, integrating modern BI solutions, such as Power BI, can provide a more efficient and robust alternative to quickly view relationships across different data streams.

## 5. Discussion

Routine behavior monitoring is becoming an integral activity of animal organizations seeking to proactively evaluate animal welfare. Behavior monitoring programs make it possible to identify meaningful changes in an individual’s baseline behavior pattern, as well as evaluate the impact of unexpected events. New data collection options, such as the ZooMonitor app, have made behavior monitoring accessible to many organizations that care for animals. Unfortunately, the dynamic nature of ongoing behavioral monitoring can present unique challenges to data analysis that may require new tools and skills. The increasing accessibility of BI tools introduces new options for managers and scientists in zoos, aquariums, and laboratories to quickly visualize behavior data to support evidence-based decision making.

While efforts to observe animal behavior are widespread in zoos and aquariums and the use of technology has been growing [33], the use of BI tools to evaluate animal behavior has, in our experience, been rare. However, some notable examples exist. At Lincoln Park Zoo, ongoing behavior data recorded by trained zoo volunteers using the ZooMonitor app are visualized using reports in Microsoft Power BI. Each species in the monitoring program has a custom-built report that provides a general summary of behavior and includes specific behavioral questions for that species. These reports are automatically refreshed weekly and are made available to curators and managers online through the Power BI service. Recently, Sea World (Australia) developed a similar process in Power BI to quickly visualize their behavior data recorded using the ZooMonitor app [34]. Shedd Aquarium has used the ZooMonitor app to record detailed 24 h data on newborn cetacean calves. These data are then visualized through Power BI reports that the managers check daily, allowing them to rapidly respond to any unexpected changes [35]. At Dallas Zoo, staff have used Power BI to track elephant activity and social behavior by visualizing the real-time locations of their elephants recorded using RFID sensors [36,37]. These examples highlight some of the potential for BI software to help address challenges in analyzing ongoing behavioral data, to address both broad and specific questions.

Although technology and advanced analytics are still nascent for many zoos and aquariums, their implementation and use have been embraced in the agriculture industry. Leveraging the potential of cameras and sensors, farmers have increasingly begun to adopt automated real-time monitoring systems to track behavioral and environmental data, a practice known as Precision Livestock Farming [38]. Schillings et al. [39] provide a review of the different parameters monitored. These have included a range of behaviors, such as general activity levels, agonistic behaviors, social interactions, and reproductive behaviors, as well as more health-focused parameters such as lameness, physical appearance, and body condition, and environmental parameters like air quality and temperature. Although these innovations have often been driven by a focus on production, their potential to impact welfare is clear [39].

Precision livestock farming has been described to be part of the fourth agricultural revolution, or Agriculture 4.0, a term referencing the Industry 4.0 movement born from the increased use of sensors, Internet of Things devices, big data, and artificial intelligence in the industrial sector [40,41]. Given this shared lineage with traditional business applications, it is not surprising that BI tools have played a key role in addressing this new torrent of data resulting from the adoption of smart farming methods [42,43,44]. These real-time monitoring systems include tools, often referred to as agricultural decision support systems, that can alert farmers to concerns and display patterns in the data through custom dashboards [44,45,46]. It has been argued that these technologies are allowing farmers to build “digital representations” of their animals (Norton et al., 2019). While the realization of a similar smart zoo concept is likely to be some years away, the recent study by Zuerl et al. [47] developed algorithms for automated video-based monitoring of polar bears at Nuremberg Zoo, which is an exciting development that hints at a future with more robust data collection than has hitherto been possible. The growth of automated monitoring through sensors and cameras in zoos has the potential to transform our ability to proactively monitor welfare [33] beyond our current limitations in staff availability and schedules, including during overnight time periods when staff are not present and live observations are impractical, but we will need corresponding advances in our analytics capabilities to harness these technologies. Previous calls have been made for greater integration of farm animal welfare science into zoo animal welfare science [48]. To add to those calls, increased attention toward techniques for analyzing data across the two disciplines may also be warranted.

Business intelligence software represents an exciting new class of tools for zoos and aquariums looking to develop ongoing behavioral monitoring programs. Adopting new technologies can be difficult but it is my hope that this guide serves as an approachable introduction to those interested in BI solutions. In addition, the Microsoft Power BI templates available to download (see Supplementary Materials S1 and S3) can provide the reader with a starting point to enhance their data visualizations and serve as a foundation for their own additional custom-built reports. Effectively using data to support evidence-based decision making can be challenging, but continued attention, research, and resources will help lead zoos and aquariums to reach their full potential as science-based organizations.

## 6. Conclusions

Animal welfare is a dynamic process and our methods to evaluate welfare and make data-driven decisions to enhance welfare must also be equally dynamic. As technology continues to evolve and unlock new opportunities for data collection, the potential for proactive animal welfare monitoring will increase, but its full potential will hinge on the ability to quickly ingest and interpret large amounts of data. The inclusion of BI tools in welfare monitoring and reporting processes can greatly enhance the impact of these activities and help foster a culture of evidence-based decision-making.

## Data Availability

Example data for use with the provided templates can be found in the Supplementary Files.

## Supplementary Materials

The following supporting information can be downloaded at: https://www.mdpi.com/article/10.3390/ani12131606/s1, File S1: Quarterly Behavior Report Template v 1.0; File S2: Example ZooMonitor Data for Quarterly Report; File S3: Enrichment Report Template v1.0; File S4: Example ZooMonitor Data for Enrichment Report; and File S5: Enrichment Report Instructions.
