# Supplementary material for: Power Up: Combining Behavior Monitoring Software with Business Intelligence Tools to Enhance Proactive Animal Welfare Reporting
Source: Animals (Basel). 2022 Jun 22;12(13):1606. doi: 10.3390/ani12131606 (PMC9264768; doi:10.3390/ani12131606)
Supplement: Supplementary file 1 [file animals-12-01606-s001.zip › animals-1760457-supplementary/Updated Supplementary Materials/S5 Enrichment Report Instructions.pdf]

Enrichment Report Instructions

Template Ver. 1.0

Step 1

To use the Enrichment Report Template, you will first need to add two behaviors to your ZooMonitor project: *Enrichment Available* and *Enrichment Interaction*. These behaviors will need to be added to an All Occurrence channel on your project. You can have additional All Occurrence behaviors added to your project and other behavior channels as needed but data from these behaviors will not be available in the Enrichment Report.

When adding the Enrichment Available behavior to your project, you will need to specify your enrichment choices as your first behavior modifier through a comma-separated value list. Please see the screenshot below for an example.

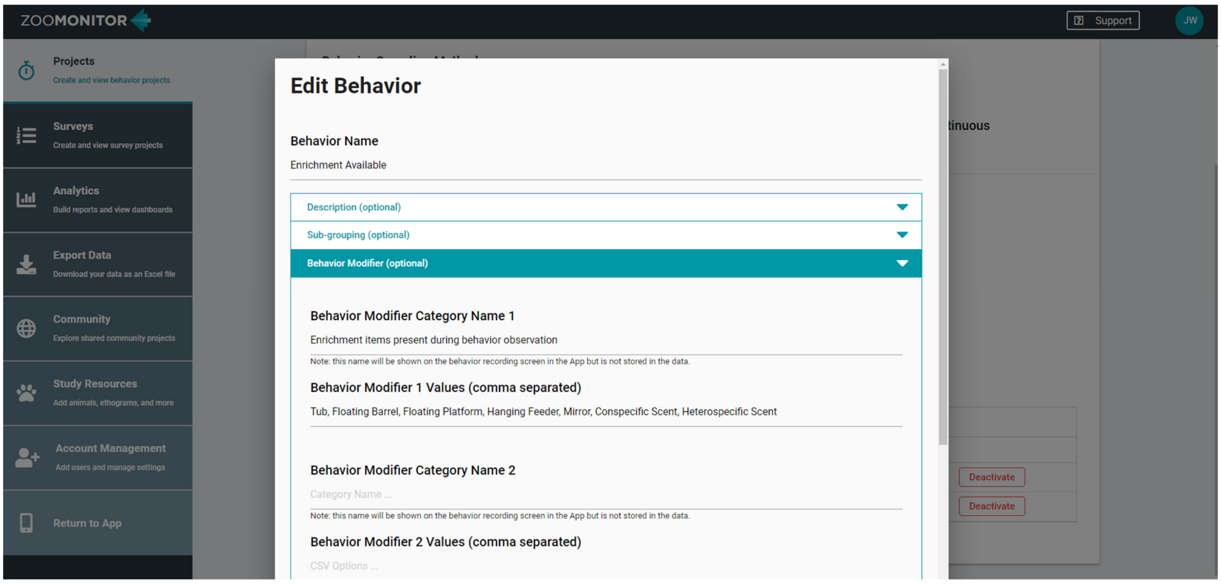

When adding the Enrichment Interaction behavior, you will need to configure three behavior modifiers: the enrichment item list as comma-separated values (be sure to match the spelling of the items you included in the Enrichment Available behavior noted above), the list of possible interaction behaviors as a comma-separated value list, and whether the behavior elicited was a goal behavior with choices of Yes, No, and NA if you do have goal behaviors specified in your enrichment evaluation program.

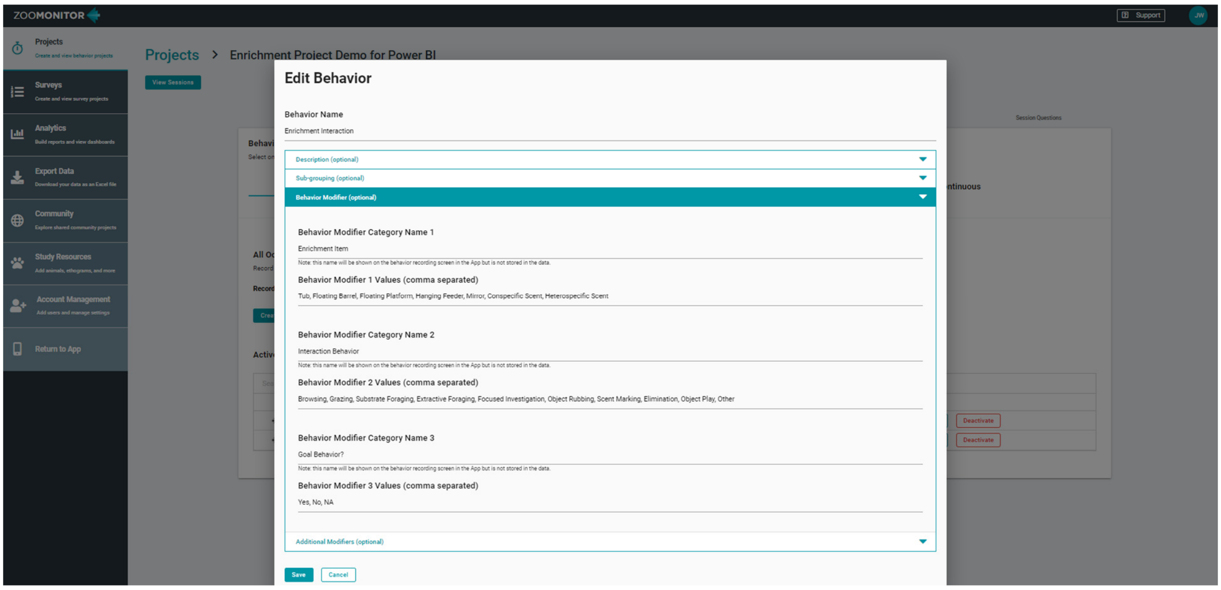

Step 2

Once you have added these behaviors and recorded data in ZooMonitor, you're ready to export these data from your ZooMonitor admin area. Your data will export as a CSV file. For use with the Enrichment Report Template, you will need to rename the tab with your data as "Raw Data" and save the file as an Excel workbook (.xls or .xlsx file format).

Step 3

Once you open the Enrichment Report Template in Power BI, you will be prompted to enter a file pathway for your data file. If using Windows, you can right click the file and select "Copy as path", then paste this pathway into the file pathway field in Power BI. Be sure to remove any quotes around your file pathway before loading the data.
